# Statistical damage model of rock considering pore water pressure and multifractal characteristics

**DOI:** 10.1371/journal.pone.0358254

**Published:** 2026-09-15

**Authors:** Yonghui Zhang, Fangtao Li

**Affiliations:** 1 Guizhou Communications Polytechnic University, Guiyang, China; 2 The Finance Bureau of Suihua City, Heilongjiang Province, Suihua, China; Northeastern University, CHINA

## Abstract

A statistical damage model incorporating multifractal characteristics is proposed for micro-fractured rocks subjected to pore water pressure. Based on the assumption that microelement strength follows a Weibull distribution, the unified strength theory is adopted as the statistical variable, and a relative fractal dimension λ is introduced to quantitatively characterize the heterogeneity and anisotropy of the material, thereby establishing a statistical damage model for the rock, which is finally validated through experiments. The results show that: (1) The model predictions agree well with test curves, effectively reflecting the mechanical responses under varying pore water pressures; (2) Increasing pore water pressure accelerates damage evolution, lowers the critical damage threshold, and promotes rock failure; (3) A larger relative fractal dimension λ reduces microelement strength concentration, brittleness, damage threshold, and overall strength, making the rock more prone to instability. This study provides a reference for safety analysis of rock engineering under stress‑seepage coupling conditions.

## 1 Introduction

In deep underground engineering, water-rich environments are frequently encountered. Under the coupled effects of water and stress, the failure process of rocks becomes considerably complex. Moreover, the presence of joints, fractures, and other defects within rocks leads to pronounced heterogeneity and nonlinearity, rendering traditional continuum constitutive models inadequate for accurately describing their failure behavior. Therefore, establishing a damage constitutive model that properly reflects hydromechanical coupling effects and the structural characteristics of rocks is of great theoretical significance and engineering value for the stability assessment of deep rock mass engineering [1].

Statistical damage theory provides an effective framework for describing the progressive failure process of rocks. Lemaitre [2] proposed the strain equivalence hypothesis, which laid the foundation for the quantitative relationship between the damage variable and effective stress. Krajcinovic et al. [3] further combined continuum damage theory with statistical strength theory, establishing the basic framework of statistical damage mechanics. Subsequently, numerous scholars have developed a series of statistical damage models for rocks along this line of research, with the research focus gradually expanding from single-factor to multi-factor coupling. For instance, Peng et al. [4] established a statistical damage model for mudstone considering strain-rate effects based on a modified Hoek-Brown criterion, and found that the influences of confining pressure and strain rate on strength are independent of each other. Liu et al. [5] established an anisotropic statistical damage model for rock under true triaxial compression based on the statistical damage theory and energy equivalence hypothesis. They found that *D*_3_ (in the direction of the maximum principal stress) dominates the anisotropic damage evolution, and the peak strength increases with the increase of the intermediate principal stress coefficient *b*. Jiang et al. [6] proposed a mechanical damage model for the entire failure process of frozen rocks and systematically analyzed the effects of confining pressure and low temperature on the brittle-ductile transition of frozen rocks. Ren et al. [7] coupled damage with plastic internal variables to establish a damage model for porous rocks applicable to different stress paths. In the field of hydromechanical coupling effects, Tan et al. [8] found through hydromechanical coupling triaxial tests that pore water pressure most significantly weakens the damage stress, and accordingly proposed two types of damage constitutive models. Yong et al. [9] simulated the acidic karst erosion environment and established a damage model incorporating chemical damage, mechanical damage, and pore water pressure coupling. Zhang et al. [10] conducted systematic experimental studies and damage constitutive modeling on multi-shape fractured sandstone under hydromechanical coupling. Wei et al. [11] proposed a new statistical damage constitutive model based on the Hoek–Brown criterion that considers the water-weakening effect. Gao et al. [12] established a wellbore-reservoir-thermo-hydro-mechanical-diffusion fully coupled model, providing important theoretical foundations for the systematic mathematical description of multi-field coupling.

The above studies indicate that the applicability of statistical damage models under complex stress states and environmental conditions is continuously expanding, particularly with increasing applications in hydromechanical coupling environments. However, most existing models still employ linear strength criteria to describe the strength of micro-elements, which fails to fully capture the nonlinear characteristics of rock strength and the effects of intermediate principal stress. To address this, Yu [13] proposed the unified strength theory, and numerous studies have demonstrated its applicability to various rock types and complex stress states. Li et al. [14] further developed a generalized unified strength theory and verified its universality through true triaxial test data of 14 rock types. Shen et al. [15] established a statistical damage constitutive model for embankment rocks based on the unified strength theory and Weibull distribution, while Cui et al. [16] applied it to the stability analysis of deep circular tunnels. Nevertheless, research incorporating the unified strength theory into hydromechanical coupling conditions for statistical damage model construction and micro-element strength distribution description remains quite limited. Furthermore, natural rocks contain intricate networks of micro-cracks and exhibit significant anisotropy, and few studies have incorporated the inherent heterogeneity of rocks into the quantitative description framework of statistical damage models [17,18].

To address these issues, this paper is based on the unified strength theory. Considering the multifractal characteristics of pore water pressure and rock material anisotropy, and assuming micro-element strength follows a Weibull distribution, a rock damage model is established. A calculation method for the model parameters is provided. Finally, the model is validated through experiments, and the influences of pore water pressure and relative fractal dimension on rock damage evolution are analyzed.

## 2. Construction of a statistical damage model of rock under water pressure

### 2.1 Model assumptions

The statistical damage model established in this paper is mainly based on the following three fundamental assumptions:

(1) The strength of rock microelements follows the Weibull statistical distribution.
(2) The relationship between nominal stress and effective stress is established using Lemaitre's strain equivalence hypothesis.

### 2.2 Damage constitutive relationship

Assuming that the failure of rock micro-elements is random, according to continuum damage mechanics, the damage variable *D* is defined as the ratio of the number of micro-elements *n* at a given stress level to the total number of micro-elements *N* in the initial state, namely:


$$\begin{array}{c} D=\frac{n}{N} \end{array}$$ (1)

It is worth noting that the total number of micro-elements *N* is assumed to be sufficiently large to satisfy the requirement of statistical stability.

According to Lemaitre's strain equivalence principle, the damage relationship of rock can be expressed as [19].


$$\begin{array}{c} {\overset{―}{\sigma }}_{ij}^{*}=\frac{\sigma_{ij}}{1-D} \end{array}$$ (2)

Where ${\overset{―}{\sigma }}_{ij}^{*}$represents the effective stress tensor borne by the material; *σ*_ij_ is the nominal stress tensor borne by the material; and *D* denotes the damage variable.

When materials are subjected to pore water pressure, the effective stress principle is employed to analyze stress-seepage coupling problems based on porous media elasticity theory. Since natural rock materials are not isotropic, a relative fractal dimension *λ* is introduced in this paper to characterize the heterogeneity and anisotropy of the rock material [18]. Based on existing research, Equation (2) is modified, and thus the effective stress tensor under the coupling effect of seepage and stress can be expressed as:


$$\begin{array}{c} \sigma_{ij}^{*}=\frac{\sigma_{ij}-p_{w}\delta_{ij}}{1-\lambda D} \end{array}$$ (3)

where σ^*^_ij_ is the effective stress tensor under stress-seepage coupling; pw is the pore water pressure; *λ* is the relative fractal dimension of the rock, where 0 < *λ* < 1. When *λ* approaches 1, it indicates the generation of infinite microcracks, and when λ approaches 0, it indicates no generation of microcracks; *δ*_ij_ is the Kronecker delta, and when *i* = *j*, *δ*_ij_ = 1.

### 2.3 Rock damage constitutive relationship based on Weibull distribution

The statistical damage constitutive model is established by integrating continuum damage theory with statistical strength theory, based on the concepts of effective stress and the assumption of strain equivalence. Among the existing distribution types for rock micro-element strength, the Weibull distribution has been widely adopted. Assuming that the strength of rock micro-elements follows a Weibull distribution, its probability density function is given by [20,21]:


$$\begin{array}{c} P\left(F\right)=\frac{m}{F_{\text{0}}}{\left(\frac{F}{F_{\text{0}}}\right)}^{m-1}exp\left[-{\left(\frac{F}{F_{\text{0}}}\right)}^{m}\right] \end{array}$$ (4)

where *P*(*F*) is the distribution function of the rock micro-element strength; *F* is the distribution variable for the random distribution of micro-element strength; and *m* and *F*_0_ are the Weibull distribution parameters, where *m* is the shape parameter reflecting the degree of concentration of microelement strength distribution(a larger *m* indicates more homogeneous material)

When loaded to a certain stress level *F*, the number of damaged micro-elements is


$$\begin{array}{c} n=\int_{\text{0}}^{F}NP\left(x\right)dx=N\left\{1-exp\left[-{\left(\frac{F}{F_{\text{0}}}\right)}^{m}\right]\right\} \end{array}$$ (5)

Substituting Equation (5) into Equation (1), then we have


$$\begin{array}{c} D=1-exp\left[-{\left(\frac{F}{F_{\text{0}}}\right)}^{m}\right] \end{array}$$ (6)

In triaxial experiments, the nominal stresses *σ*_1_, *σ*_2_, *σ*_3_ and the axial strain *ε*_1_ can be easily obtained. Before the rock reaches the damage threshold *ε*_f_, assuming that the deformation of σ*_1_ obeys the linear elastic Hooke's law and satisfies the deformation compatibility condition (*ε*_1_ = ε^*^_1_), we can get:


$$\begin{array}{c} \varepsilon_{\text{1}}^{*}=\varepsilon_{\text{1}}=\frac{\text{1}}{E}\left[\sigma_{\text{1}}^{*}-\mu \left(\sigma_{\text{2}}^{*}+\sigma_{\text{3}}^{*}\right)\right] \end{array}$$ (7)

where *μ* is the Poisson's ratio, and *σ*_1_, *σ*_2_, *σ*_3_ satisfy Equation (3). Expressing *σ*_1_, *σ*_2_, *σ*_3_ using Equation (3) which includes the pore water pressure *p*_*w*_, we can obtain:


$$\begin{array}{c} \left\{ \begin{array}{c} @l@\sigma_{\text{1}}^{*}=\frac{\sigma_{\text{1}}-p_{w}}{1-\lambda D}\mathrm{\backslash vspace}1mm\\ \sigma_{\text{2}}^{*}=\frac{\sigma_{\text{2}}-p_{w}}{1-\lambda D}\mathrm{\backslash vspace}1mm\\ \sigma_{\text{3}}^{*}=\frac{\sigma_{\text{3}}-p_{w}}{1-\lambda D} \end{array}\right. \end{array}$$ (8)

Substituting the above equation (8) into equation (7) gives


$$\begin{array}{c} \sigma_{\text{1}}=(1-\lambda D)E\varepsilon_{\text{1}}+\mu (\sigma_{\text{2}}+\sigma_{\text{3}})+p_{w}(1-2\mu ) \end{array}$$ (9)

Finally, by substituting equation (6) into equation (9), the statistical strength constitutive relationship of rock damage based on the Weibull distribution under the consideration of pore water pressure can be obtained.


$$\begin{array}{c} \sigma_{\text{1}}=E\varepsilon_{\text{1}}\left\{1-\lambda +\lambda exp\left[-{\left(\frac{F}{F_{\text{0}}}\right)}^{m}\right]\right\}+\mu (\sigma_{\text{2}}+\sigma_{\text{3}})+p_{w}(1-2\mu ) \end{array}$$ (10)

### 2.4 Statistical distribution variables of rock microelements based on the unified strength theory

The unified strength theory is a collection of a series of yield criteria and failure criteria, characterized by a unified mechanical model, unified mathematical modeling equations, and unified mathematical expressions, making it applicable to various different materials [13,22]. This strength theory addresses the shortcomings of traditional Mohr-Coulomb and Hoek-Brown strength criteria. For rock materials, with compressive stress defined as positive and tensile stress as negative, and assuming the principal stress order as *σ*_1_ ≥ *σ*_2_ ≥ *σ*_3_, the expression of the unified strength theory is given as follows:


$$\begin{array}{c} \left\{ \begin{array}{c} @l@F=\sigma_{\text{1}}^{*}-\frac{(1+sin\varphi )(b\sigma_{\text{2}}^{*}+\sigma_{\text{3}}^{*}\text{)}}{(1+b)(1-sin\varphi \text{)}}=\frac{2ccos\varphi }{1-sin\varphi }\\ \left(\sigma_{\text{2}}^{*}\leq \frac{\sigma_{\text{1}}^{*}+\sigma_{\text{3}}^{*}}{\text{2}}+\frac{\sigma_{\text{1}}^{*}-\sigma_{\text{3}}^{*}}{\text{2}}sin\varphi \right)\\ F=\frac{\sigma_{\text{1}}^{*}+b\sigma_{\text{2}}^{*}}{1+b}-\frac{(1+sin\varphi )\sigma_{\text{3}}^{*}}{1-sin\varphi }=\frac{2ccos\varphi }{1-sin\varphi }\\ \left(\sigma_{\text{2}}^{*}\geq \frac{\sigma_{\text{1}}^{*}+\sigma_{\text{3}}^{*}}{\text{2}}+\frac{\sigma_{\text{1}}^{*}-\sigma_{\text{3}}^{*}}{\text{2}}sin\varphi \right) \end{array}\right. \end{array}$$ (11)

Where *c* is the cohesion of the rock material; *φ* is the internal friction angle of the rock material; and *b* is the intermediate principal stress coefficient, with 1 ≥ *b* ≥ 0. When *b* = 0, it corresponds to the Mohr-Coulomb strength criterion; when *b* = 1, it corresponds to the twin shear strength criterion.

By substituting equation (8) into equation (11), the expression for the distribution variable of rock microelement strength can be obtained.


$$\begin{array}{c} F=\frac{\text{1}}{1-\lambda D}\left\{\sigma_{\text{1}}-p_{w}-\frac{(1+sin\varphi )\left[b(\sigma_{\text{2}}-p_{w})+\sigma_{\text{3}}-p_{w}\right]}{(1+b)(1-sin\varphi \text{)}}\right\} \end{array}$$ (12)

Among them, 1-*λD* can be obtained by transforming equation (9). Express 1-*λD* using equation (9) and substitute it into equation (12) to obtain:


$$\begin{array}{c} F=\frac{E\varepsilon_{\text{1}}(\sigma_{\text{1}}-p_{w})}{\sigma_{\text{1}}-\mu (\sigma_{\text{2}}+\sigma_{\text{3}})-p_{w}(1-2\mu )}-\frac{E\varepsilon_{\text{1}}(1+sin\varphi )\left[b(\sigma_{\text{2}}-p_{w})+\sigma_{\text{3}}-p_{w}\right]}{\left[\sigma_{\text{1}}-\mu (\sigma_{\text{2}}+\sigma_{\text{3}})-p_{w}(1-2\mu )\right](1+b)(1-sin\varphi \text{)}} \end{array}$$ (13)

Finally, by substituting equation (13) into equation (10), the statistical damage model of rock based on the unified strength theory can be obtained.

## 3 Determination and validation of model parameters

### 3.1 Determination of model parameters

Existing studies generally determine the parameters *m* and *F*_0_ in the constitutive equation (9) through fitting experimental data. However, this method is relatively cumbersome, and the experimental data often exhibit significant discreteness, leading to considerable influence from human factors on the results [23,24]. To avoid interference from human factors, the model parameters can be determined by utilizing the characteristic that the slope of the rock stress-strain curve is zero at its peak [25].

First, compute the partial derivative of *F* with respect to strain *ε*_1_ using equation (13), thereby obtaining:


$$\begin{array}{c} \frac{\partial F}{\partial \varepsilon_{\text{1}}}=\frac{F}{\varepsilon_{\text{1}}}=\frac{E(\sigma_{\text{1}}-p_{w})}{\sigma_{\text{1}}-\mu (\sigma_{\text{2}}+\sigma_{\text{3}})-p_{w}(1-2\mu )}-\frac{E(1+sin\varphi )\left[b(\sigma_{\text{2}}-p_{w})+\sigma_{\text{3}}-p_{w}\right]}{\left[\sigma_{\text{1}}-\mu (\sigma_{\text{2}}+\sigma_{\text{3}})-p_{w}(1-2\mu )\right](1+b)(1-sin\varphi \text{)}} \end{array}$$ (14)

Assume that the peak stress and its corresponding strain of the rock under pore water pressure are *σ*_c_ and *ε*_c_, respectively, with the corresponding peak point of the stress-strain curve being *c.* Substituting equation (13) into equation (10) and then taking the partial derivative with respect to *ε*_1,_and combining with equation (14) after simplification, we obtain:


$$\begin{array}{c} \frac{\partial \sigma_{\text{1}}}{\partial \varepsilon_{\text{1}}}=E\left\{1-\lambda +\lambda exp\left[-{\left(\frac{F}{F_{\text{0}}}\right)}^{m}\right]\right\}-\lambda mEexp\left[-{\left(\frac{F}{F_{\text{0}}}\right)}^{m}\right]{\left(\frac{F}{F_{\text{0}}}\right)}^{m} \end{array}$$ (15)

When the rock stress-strain curve reaches the peak point *c*, its slope is 0. Substituting this condition into the above equation (15) yields:


$$\begin{array}{c} exp\left[-{\left(\frac{F_{c}}{F_{\text{0}}}\right)}^{m}\right]=\frac{\lambda -1}{\lambda -m\lambda {\left(\frac{F_{c}}{F_{\text{0}}}\right)}^{m}} \end{array}$$ (16)

Moreover, since the rock stress-strain curve satisfies equation (10) at the peak point *c,* substituting the stress *σ*_c_ at the peak point and the distribution variable *F*_c_ for the random distribution of microelement strength into equation (10) yields:


$$\begin{array}{c} \sigma_{c}=E\varepsilon_{c}\left\{1-\lambda +\lambda exp\left[-{\left(\frac{F_{c}}{F_{\text{0}}}\right)}^{m}\right]\right\}+\mu (\sigma_{\text{2}}+\sigma_{\text{3}})+p_{w}(1-2\mu ) \end{array}$$ (17)

Transforming the above equation (17) gives:


$$\begin{array}{c} exp\left[-{\left(\frac{F_{c}}{F_{\text{0}}}\right)}^{m}\right]=\frac{\sigma_{c}-\mu (\sigma_{\text{2}}+\sigma_{\text{3}})-p_{w}(1-2\mu )}{\lambda E\varepsilon_{c}}-\frac{1-\lambda }{\lambda } \end{array}$$ (18)

By simultaneously solving equations (16), (17), and (18), the distribution parameter *m* of the rock microelement strength can be determined. Here, *m* is given by:


$$\begin{array}{c} m=\frac{\frac{(\lambda -1)E\varepsilon_{c}}{\sigma_{c}+(\lambda -1)E\varepsilon_{c}-\mu (\sigma_{\text{2}}+\sigma_{\text{3}})-p_{w}(1-2\mu )}-1}{\text{In}\frac{\sigma_{c}+(\lambda -1)E\varepsilon_{c}-\mu (\sigma_{\text{2}}+\sigma_{\text{3}})-p_{w}(1-2\mu )}{\lambda E\varepsilon_{c}}} \end{array}$$ (19)

Then, by substituting equation (19) back into equation (17), the distribution variable *F*_0_ for the random distribution of rock microelement strength can be obtained. *F*_0_ is calculated as:


$$\begin{array}{c} F_{\text{0}}=\frac{F_{c}}{\sqrt[{m}]{\frac{\lambda E\varepsilon_{c}}{\sigma_{c}+(\lambda -1)E\varepsilon_{c}-\mu (\sigma_{\text{2}}+\sigma_{\text{3}})-p_{w}(1-2\mu )}}} \end{array}$$ (20)

### 3.2 Model validation

To validate the proposed model, conventional triaxial pore-water pressure tests were conducted on dolomitic limestone samples collected from the Liaoshan Tunnel on the Emei–Hanyuan Expressway in Sichuan Province, China. This tunnel is characterized by well‑developed karst features, complex hydrogeological conditions, and locally high water pressure, making it a typical high‑stress, high‑water‑pressure tunnel project in southwestern China. The selection of this lithology for experimental investigation holds direct practical significance for guiding the design and construction of tunnel engineering under such complex geological conditions.

The tests were performed using the SAS‑2000 multi‑field coupled dynamic disturbance triaxial rheological testing system for rock mass, manufactured by Changchun Xinte Testing Machine Co., Ltd. Its main technical specifications are: axial force ≤ 2700 kN, confining pressure ≤ 50 MPa, and pore water pressure ≤ 50 MPa. When applying confining and pore pressures to the specimens, the loading rate of pore water pressure was set slightly lower than that of confining pressure to prevent seal failure caused by instantaneous pore pressure exceeding the confining pressure. The rock samples were machined into cylindrical specimens with a diameter of 50 mm and a height of 100 mm (Φ50 × 100 mm). The confining pressure was set at 8 MPa, and the pore water pressures were 0 MPa, 1 MPa, 3 MPa, and 5 MPa, respectively. During the testing procedure, the confining pressure was first applied at a rate of 0.5 MPa/s; after the confining pressure stabilized, the axial load was then applied at an axial displacement rate of 0.1 mm/s.

**Fig 1:**
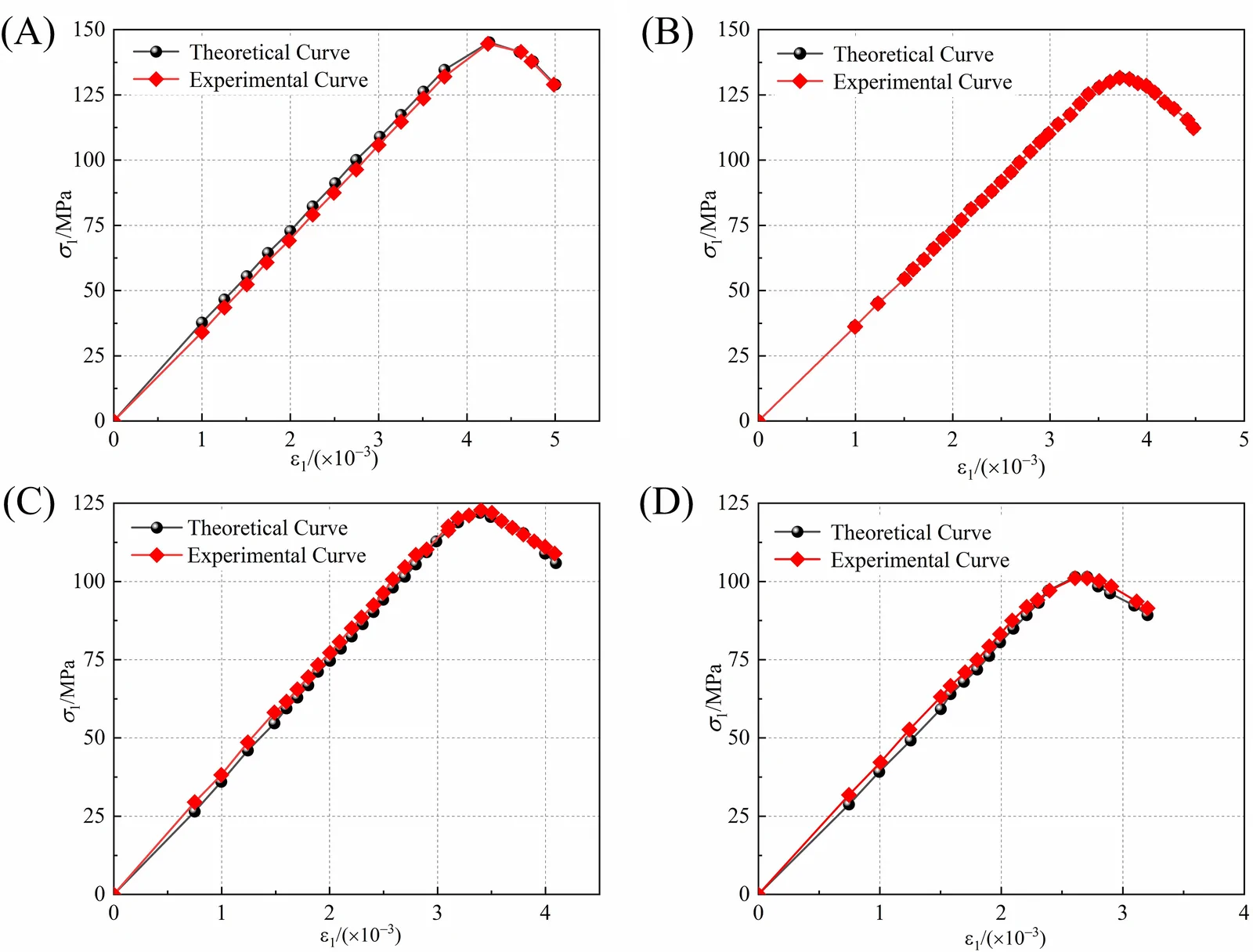
Comparison between test and theoretical curves. (A) *p*_*w*_ = 0MPa.(B) *p*_*w*_ = 1MPa.(C) *p*_*w*_ = 3MPa.(D) *p*_*w*_ = 5MPa.

The Poisson's ratio of the dolomitic limestone obtained from the tests was 0.24, and its internal friction angle was 34.3°. The model parameters calculated based on Equations (19) and (20) in this paper, along with the experimental data, are shown in Table 1. Using these parameters, the theoretical stress-strain relationship curves of the rock under different pore water pressures were calculated via Equation (10) and compared with the experimental curves, as illustrated in Fig 1.

**Table 1:** Model parameters.

*p*_*w*_/MPa	*E*/GPa	*m*	*F*_0_/MPa
0	35.5	14.1	153.58
1	36.8	17.8	118.02
3	38.7	20.9	91.74
5	41.6	23.9	67.07

As can be seen from Fig 1, the curves of the statistical damage model for rock considering pore water pressure and multifractal characteristics align well with the experimental curves. Table 1 indicates that as the pore water pressure increases, the model parameter *m* gradually increases while *F*_0_ gradually decreases, which is consistent with the established patterns of statistical damage model parameters for rocks [26].The parameter *m* reflects the concentration degree of the microelement strength distribution of the rock; a larger *m* indicates that, with increasing pore water pressure, the distribution of rock microelement strength becomes more concentrated, and the brittleness of the material increases. The parameter *F*_0_ represents the macroscopic strength of the rock; a larger *F*_0_ indicates greater macroscopic strength of the rock. From Table 1 and Fig 1, it can be observed that *F*_0_ gradually decreases as the pore water pressure increases. This is consistent with the rule that rock strength decreases with increasing water pressure under stress-permeability coupling effects.

## 4 Relationship between pore water pressure, relative analysis dimension, and damage characteristics

### 4.1 Relationship between pore water pressure and damage evolution

**Fig 2:**
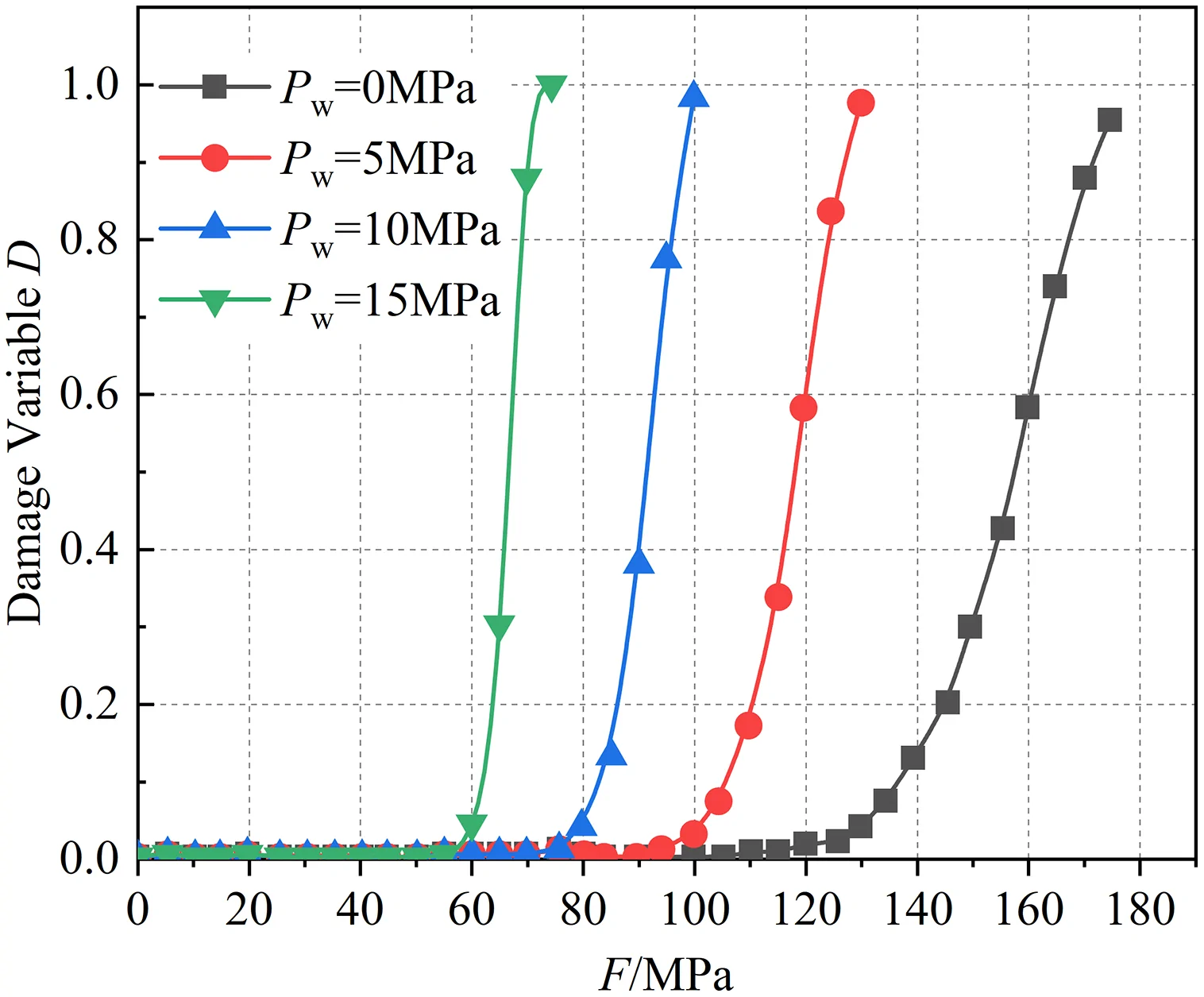
Damage variable *D* value under different pore water pressure.

In practical engineering, when water pressure is present, analyzing the safety of rock under stress-seepage coupling is of significant importance. To study the damage evolution of rock under pore water pressure, the damage variable evolution under different water pressures was calculated based on Equation (6) for the tested surrounding rock at 5 MPa, as shown in Fig 2. From Fig 2, it can be observed that as the water pressure gradually increases, the damage variable of the rock increases more rapidly, and the slope of the curve becomes steeper. This indicates that the increase in pore water pressure accelerates the damage evolution of the rock. In actual tunnel engineering, implementing drainage and waterproofing measures in advance contributes to the stability of tunnel surrounding rock and the safety of excavation.

The critical damage degree characterizes a parameter similar to rock strength parameters that indicates whether rock failure occurs [27]. It refers to the damage variable *D*_c_ corresponding to the strain at the peak stress of the rock, with the corresponding microelement strength at the peak stress being *F*_c._ According to the definition, the critical damage degree is given by:


$$\begin{array}{c} D_{c}=1-exp\left[-{\left(\frac{F_{c}}{F_{\text{0}}}\right)}^{m}\right] \end{array}$$ (21)

**Fig 3:**
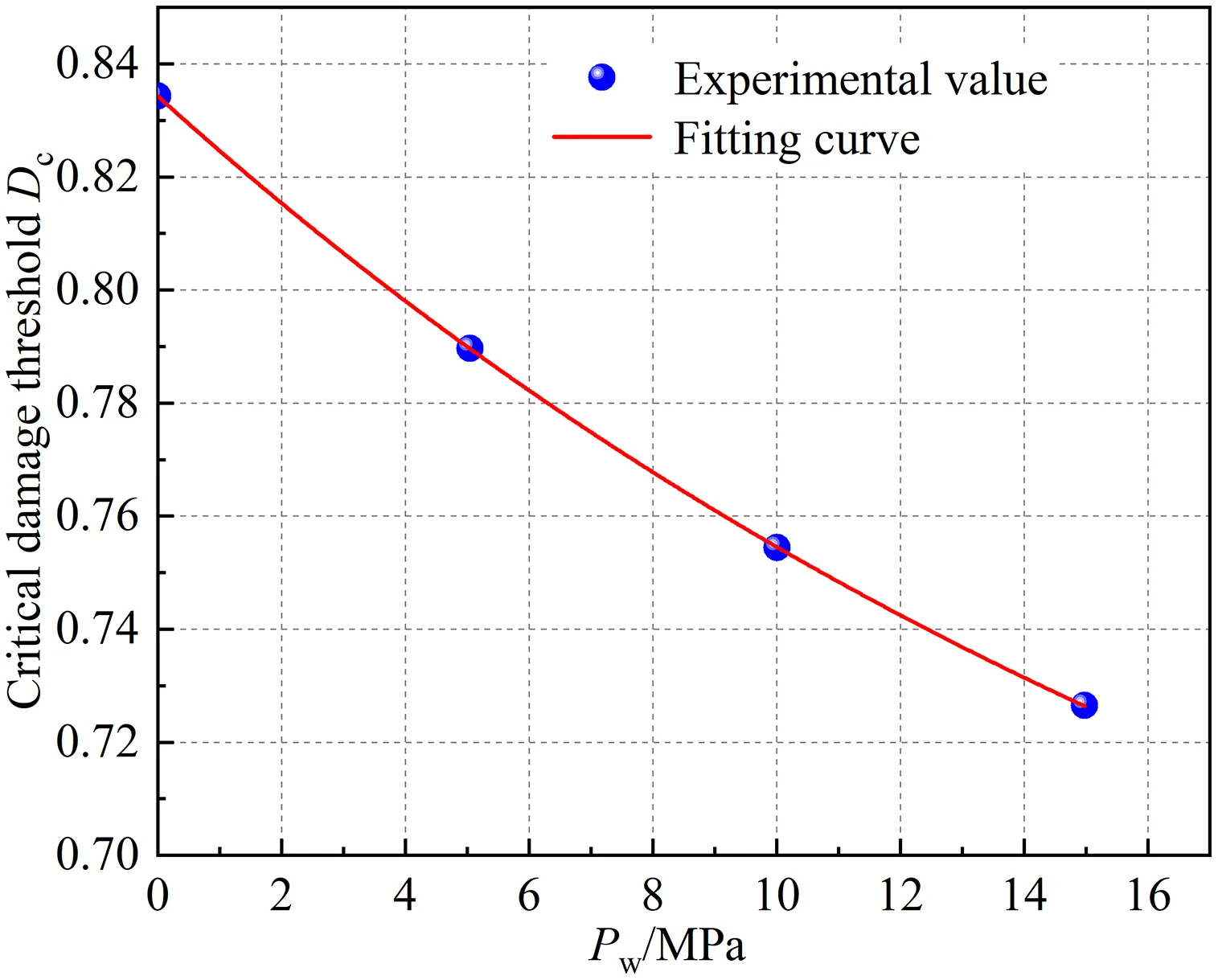
Critical damage variable *D*_c_ value under different pore water pressure.

The critical damage degree *D*_c_ values under different pore water pressures in the tests conducted in this paper were calculated based on the above equation (21), as shown in Fig 3. From the figure, it is clearly evident that as the pore water pressure increases, the critical damage degree of the rock gradually decreases. This indicates that the increase in pore water pressure leads to a gradual reduction in the damage value at which rock failure occurs, meaning that the rock becomes more prone to failure under the influence of pore water pressure.

### 4.2 Relationship between relative fractal dimension and damage evolution

**Fig 4:**
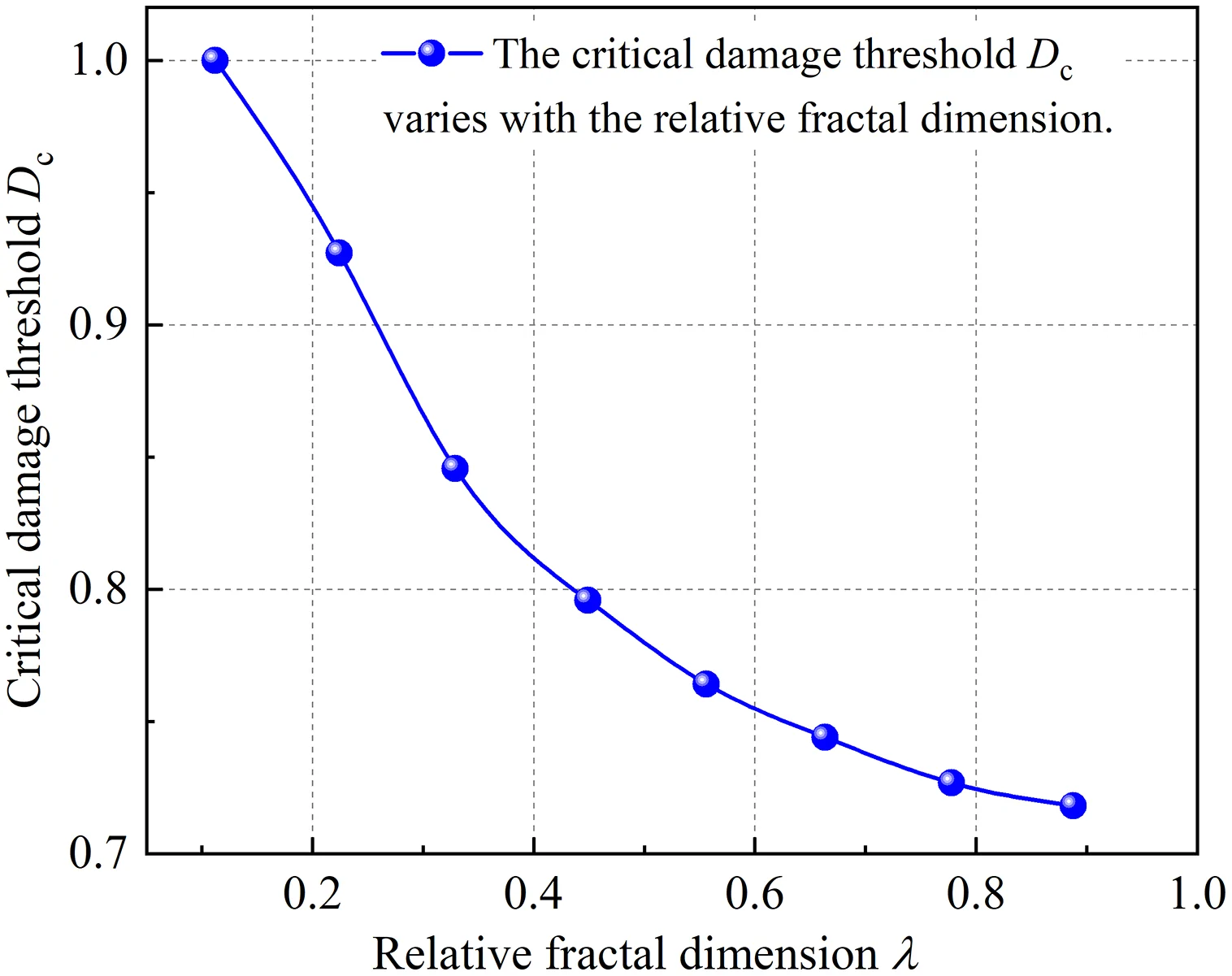
Relationship curve between critical damage variable *D*_c_ and relative fractal dimension *λ.*

**Fig 5:**
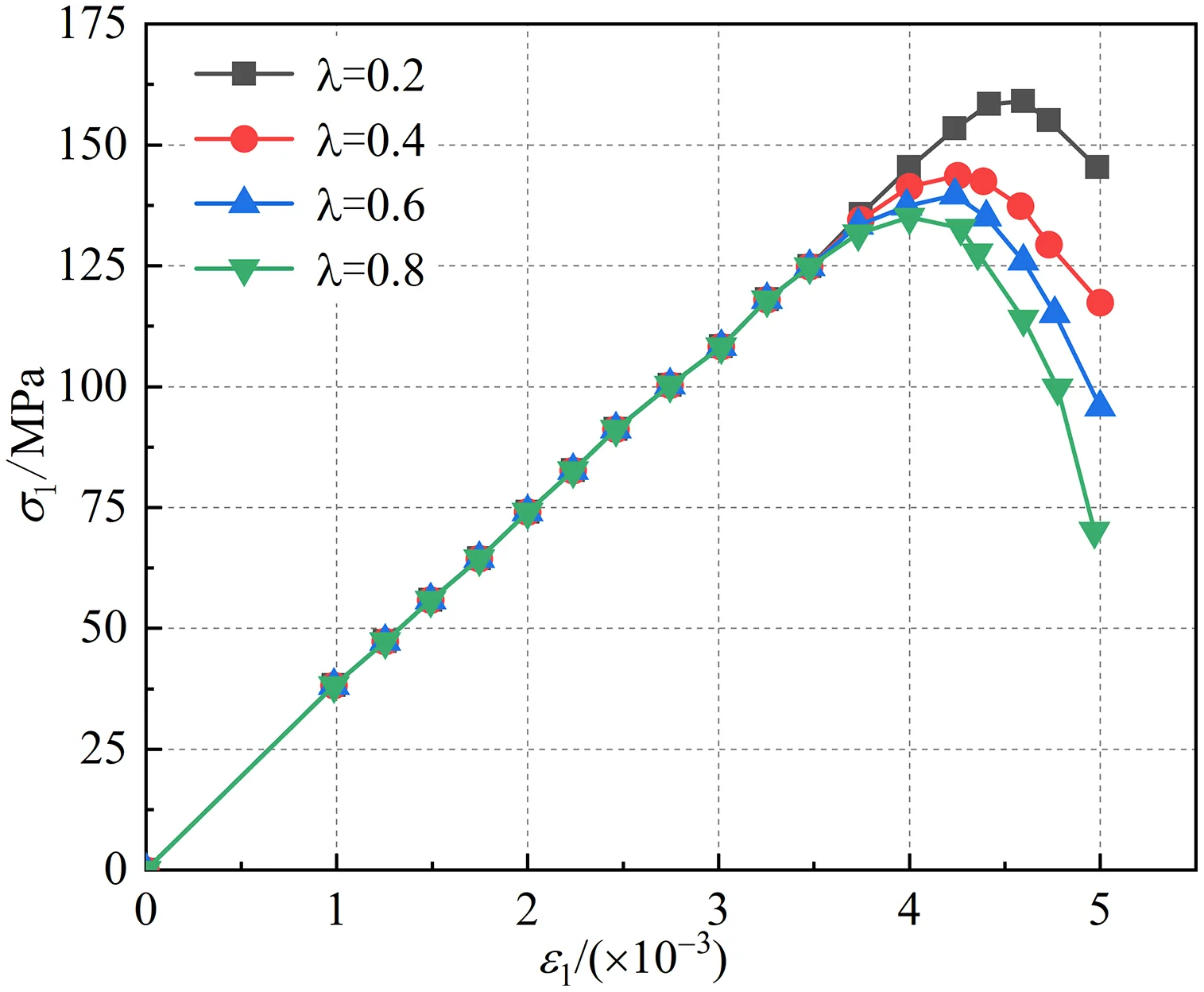
Relationship curve between stress *σ*_1_ and relative fractal dimension *λ.*

Natural rocks contain numerous intricate micro-fractures with varied phases and morphologies, and the distribution characteristics of these micro-fractures significantly influence rock strength. To analyze the effect of the relative fractal dimension of rocks on their damage evolution and strength, a set of experimental data from this study with a pore water pressure of 0 was examined. By varying the relative fractal dimension *λ,* the corresponding parameters m, *F*_c_ and *F*_0_ were determined (as shown in Table 2). Finally, the critical damage degree and stress *σ*_1_ under different relative fractal dimensions *λ* were obtained, as illustrated in Fig 4 and 5.

**Table 2:** Weibull distribution parameters under different relative fractal dimensions.

Relative fractal dimension *λ*	*m*	*F*_0_/MPa	*p*_*w*_/MPa
0.2	48.4	152.14	0
0.3	17	151.7	
0.4	14.1	153.58	
0.5	12.9	154.87	
0.6	12.3	155.76	
0.7	11.9	156.42	
0.8	11.6	156.92	
0.9	11.4	157.31	

From Table 2, it can be observed that as the relative fractal dimension *λ* increases, the Weibull parameter *m* gradually decreases, while *F*_0_ gradually increases. This indicates that with an increase in the relative fractal dimension *λ,* the concentration of rock microelement strength distribution decreases, leading to a reduction in rock brittleness and an increase in macroscopic strength. Fig 4 and 5 show that as the relative fractal dimension *λ* increases, the critical damage degree *D*_*c,*_ peak stress *σ*_c,_ and strain *ε*_c_ all gradually decrease, suggesting that both the rock damage threshold and strength diminish with an increase in the rock fractal dimension *λ.* This aligns with the established patterns observed in previous studies on Weibull parameters for rock strength with multifractal characteristics [27].

## 5 Discussion

### 5.1 Advantages and limitations of the model

Compared with existing studies [18], the proposed model achieves a quantitative expression of fractal characteristics in the statistical damage model by introducing the relative fractal dimension λ as an endogenous variable into the effective stress correction. This approach better reflects the inherent heterogeneity and anisotropy of rock materials than traditional phenomenological models. Regarding the selection of strength criteria, most existing statistical damage models for rock adopt the Mohr-Coulomb criterion [28,29] or the Drucker-Prager criterion [27]. Although both are concise in form, the former neglects the influence of the intermediate principal stress, while the latter suffers from less explicit physical meaning of its parameters. The unified strength theory adopted in this study encompasses the above criteria as special cases and achieves a unified description for different rock types and stress states, thereby offering wider applicability.

However, the proposed model does not account for residual strength in the post-peak stage. The simulation accuracy for the stress-strain curve is relatively high at the peak point and in the early post-peak softening stage, but the calculated values may be underestimated in the large-deformation residual stage. For engineering problems requiring accurate simulation of residual strength, it is advisable to introduce an independent residual strength term for modification. In addition, the model has currently been validated only under conventional triaxial compression conditions (*σ*_1_ > *σ*_2_ = *σ*_3_). Although its applicability to tensile or tensile-shear composite stress states is theoretically feasible, further investigation is still required.

### 5.2 Damage acceleration characteristics of rock mass and implications

In this study, the accelerating evolution of the damage variable *D* with the loading process (see Fig 2) is intrinsically consistent with the power-law acceleration behavior widely observed in rock failure precursor processes. Previous studies have demonstrated that acoustic emission precursor analysis based on the time-reversed Omori law can effectively achieve pseudo-real-time prediction of the failure time of brittle rocks, and that damage acceleration serves as a universal precursor to impending rock failure [30–32]. The present study further reveals that elevated pore water pressure not only reduces the critical damage degree *D*_c_, but also accelerates the approach of *D* toward *D*_c_, thereby shortening the warning time window. This finding provides a theoretical basis for the determination of dynamic early warning thresholds for surrounding rock in water-rich tunnels.

### 5.3 Engineering application implications

The proposed model provides a quantitative pathway for the safety analysis of underground engineering in high-water-pressure tunnels, specifically encompassing the following three steps:

(1) Parameter acquisition. Through conventional triaxial tests under pore water pressure, the basic mechanical parameters (*E*, *μ*, *c*, *φ*) and model parameters (*m*, *F*₀) of the target surrounding rock are obtained, and the damage constitutive equation for the rock mass is established.
(2) Monitoring inversion and early warning criterion. A strain monitoring system is installed on the tunnel wall to acquire axial strain *ε*_1_ data at key locations of the surrounding rock. The current damage variable *D* is then inversely determined in real time using Equations (9) and (12). The critical damage degree *D*_c_ determined by Equation (21) serves as the threshold for stability evaluation (with its value depending on the actual in-situ pore water pressure conditions). When *D* approaches *D*_c_, it indicates that the surrounding rock is approaching its peak bearing capacity and is about to enter the accelerated damage stage.
(3) Engineering decision-making. Upon triggering of the early warning, active control measures such as enhanced bolt-shotcrete support, advance grouting reinforcement, or drainage and pressure relief can be implemented in a timely manner. For example, when monitoring data indicate that *D* is rapidly approaching *D*_c_, priority should be given to drainage and pressure relief measures, which can effectively reduce pore water pressure, thereby raising the *D*_c_ threshold and retarding the damage evolution process. The above pathway integrates laboratory testing, theoretical modeling, and field monitoring, providing a quantitative decision-making basis for the dynamic design and information-based construction of tunnel surrounding rock.

## 6 Conclusions

A statistical damage constitutive model incorporating pore water pressure and multifractal characteristics is established based on the Weibull distribution and the unified strength theory. The main conclusions are as follows:

(1) The statistical damage model established in this study, which incorporates pore water pressure and the multifractal characteristics of rock, shows good agreement between the calculated results and the experimental curves. It effectively reflects the stress-strain relationship of rocks under different pore water pressures and with internal micro-fractures.
(2) An increase in pore water pressure promotes the damage evolution of rock. As pore water pressure rises, the critical damage degree of rock gradually decreases, making the rock more prone to failure under the influence of pore water pressure.
(3) As the relative fractal dimension of the rock increases, the concentration and brittleness of the microelement strength distribution gradually decrease, while the macroscopic strength increases. However, the rock damage threshold and strength also continuously diminish.

List of symbols

*D* The damage variable

*n* The number of damaged micro-elements

*N* The total number of micro-elements

${\overset{―}{\sigma }}_{ij}^{*}$ The effective stress tensor borne by the material

*σ*_*ij*_ The nominal stress tensor borne by the material

σ^*^_ij_ The effective stress tensor under stress-seepage coupling

*p*_w_ The pore water pressure

*λ* The relative fractal dimension of the rock

*δ*_ij_ The Kronecker delta, and when *i* = *j*, *δ*_ij_ = 1.

*P*(*F*) The distribution function of the rock micro-element strength

*F* The distribution variable for the random distribution of micro-element strength *m* The Weibull distribution parameters.

*F*_0_ The Weibull distribution parameters.

*F*_*c*_ The value of the microelement strength distribution variable at the peak stress point

*E* The elastic modulus

*μ* The Poisson's ratio

*c* The cohesion of the rock material

*φ* The internal friction angle of the rock material

*b* The intermediate principal stress coefficient

*ε*_1_ The axial strain

*σ*_1_, *σ*_2_, *σ*_3_ The nominal stresses

*σ*_c_ The peak stress of the rock under pore water pressure

*ε*_c_ The strain corresponding to the peak stress under pore water pressure.

## Supporting information

S1 File The supporting documents contain detailed figure data. (7Z)
